# A pilot evaluation of the efficacy and acceptability of a novel imaginal exposure prevention (I-ERP) group programme to treat core weight, shape and social fears or phobias in adolescents with anorexia nervosa in an inpatient setting

**DOI:** 10.1007/s40519-025-01723-6

**Published:** 2025-02-15

**Authors:** Kelsie Smith, Jessica Grant, J. Hubert Lacey

**Affiliations:** 1 Schoen Clinic Newbridge, Birmingham, UK; 2 St George’s, University of London, London, UK

**Keywords:** Anorexia nervosa, Eating disorders, Adolescents, Imaginal exposure prevention, Group therapy, Weight and shape phobia, Social anxiety, Short-term outcome, Evaluation

## Abstract

**Background:**

Anxiety due to a phobia of normal body weight is a core feature and maintenance factor of anorexia nervosa (AN). This is the first study to explore the efficacy and acceptability of using a novel imaginal exposure response prevention group to target fears associated with being a normal body weight to reduce anxiety in adolescents with AN.

**Methods:**

The lead author adapted an I-ERP manual used to treat AN in adults in 1–1 therapy. Content was adapted for an adolescent population, sessions reduced from 10 to 4 and was delivered in a group format with audio recordings to be more accessible for patients. Nineteen patients with AN completed the group and the group therapist collected outcome measures before and after the intervention. A paired samples *t*-test was used to assess change in eating disorder psychopathology (EDEQ), anxiety and depression (RCAD) and fear of food (FOFM). Qualitative feedback to assess acceptability was also gathered.

**Results:**

Statistically significant reduction in anxiety in a variety of situations pertaining to weight and shape was found after completion of the group. There were no significant changes shown in eating disorder psychopathology. Adolescents provided qualitative feedback which suggested the intervention was acceptable for users.

**Conclusions:**

I-ERP which has been adapted for adolescents with AN in a group format seems to improve eating disorder psychopathology and reduce weight, shape, social and separation anxiety and phobias when used as an adjuvant to inpatient treatment. Further controlled research is advised.

**Level of evidence:**

Level III.

**Supplementary Information:**

The online version contains supplementary material available at 10.1007/s40519-025-01723-6.

## Background

Anorexia nervosa (AN) is a psychological condition associated with a distorted body image, low weight, secondary hormonal abnormalities, and frequent mood disturbance [11]. The psychopathology is dominated by a phobic avoidance of normal body weight [9, 10, 24, 25, 34] associated with an overvaluation of shape, weight, and control [3]. In an attempt to prevent the fear associated with normal body weight, individuals develop weight avoidance behaviour by restricting food, and possibly purging, over-exercising, and laxative misuse [14]. These behaviours lead to emaciation and reinforce the irrational fears by offering short-term relief [32]. AN shares the mechanism observed in other anxiety disorders whereby avoidance prevents exposure to core fears which perpetuates anxiety and reinforces engagement in avoidance behaviours [18, 43]. Treatment [1, 7] often incorporates exposure techniques derived from behaviourism and learning theories (originally proposed by Watson [41]) to enable individuals to confront their core fears and to break the cycle of reinforcement [39].

Previous research has suggested similarities between AN and obsessive–compulsive disorder (OCD) [30]. Levinson et al. [28] stated the similarities include intrusive/fearful thoughts, the compulsion to perform rituals to reduce anxiety, and obsessions maintaining rituals. The fearful thoughts in AN relate to food and thinness [39], whereas they are more general amongst those with OCD. Exposure and response prevention (ERP) is a form of cognitive behavioural therapy (CBT) that includes psychoeducation, exposure to core fears, and prevention of performing rituals or safety behaviours and is the gold standard treatment used in OCD [23]. The National Institute for Health and Care Excellence [33] recommends ERP due to robust empirical evidence supporting its efficacy [20]. ERP enables patients to recognise and confront fears in safe environments, facilitating habituation, a process in which repeated exposure diminishes anxiety by lessening sensitivity to the feared stimulus [37, 42].

Phobic avoidance of normal body weight has been shown to occur as a result of body image disturbance [17]. ERP has previously been applied to AN to target body image disturbance through methods like mirror exposure [19], which has shown reductions in body image dissatisfaction and avoidance behaviours in women [12, 21]. Morgan et al. [31] demonstrated that a ten-session mindfulness-based CBT programme incorporating mirror exposure led to significant reductions in body-checking behaviours, body avoidance, and anxiety, while improving shape and weight concerns in individuals with AN. However, this approach may have limited effectiveness, as it primarily addresses physical stimuli and may not fully target the core fears underlying AN [40]. Imaginal exposure (IE), in which patients confront fears through mental imagery, has shown efficacy in addressing core fears in other disorders, such as PTSD and OCD [15, 16, 45]. Pittig et al. [36] described how IE works by creating associations in the brain similar to in vivo exposure. Patients learn that despite feeling anxious, exposure does not lead to their feared catastrophe. Agren et al. [2] showed that IE is as effective as in vivo exposure and activates the same brain regions in neuroimaging research.

Imaginal exposure therapy is based on the avoidance-anxiety model, where avoidance maintains anxiety. If a patient continues to avoid their fears, they do not get the opportunity to learn that they are not life-threatening. Research extending IE to AN has highlighted promising results. Levinson et al. [27] presented a case study in which a patient with AN articulated core fears about identity loss if weight was gained. Using imaginal exposure, scriptwriting, and thought processing, the intervention achieved significant reductions in disordered eating symptoms and anxiety. Levinson et al. [29] further explored IE through a 4-week online trial, where participants developed individual fear scripts and experienced significant reductions in ED symptoms, fears, and anxiety, with improvements maintained at 6-month follow-up. In a randomised controlled trial, Butler and Heimberg [4] examined IE for AN, finding it was more acceptable to participants than combined food-exposure techniques, with improvements in distress tolerance and self-efficacy. These results indicate that IE may effectively address core fears in AN by activating feared outcomes and fostering emotional habituation.

Building on this body of research, the authors felt there was evidence to suggest that imaginal exposure therapy might alleviate the core fears of anorexia nervosa in adolescence, where therapy might be more effective than in adults. This exploratory research adapted the treatment for a shorter format suitable for adolescent patients, delivered in a four-session group format to accommodate public healthcare resource limitations. In order to evaluate the acceptability of the group intervention, the therapist gathered qualitative feedback after the group was completed.

The study assesses the efficacy and acceptability of this I-ERP intervention, hypothesising reductions in ED symptoms, anxiety, and core fears from pre- to post-treatment.

## Methods

### Aim

This study aimed to explore the efficacy and acceptability of I-ERP when given in short-term group format for adolescents with AN, by comparing measures before and after the intervention, together with measuring SUDS within the sessions. Acceptability was assessed through qualitative patient feedback on the completion of the group. There was no control group included.

### Setting

The study was carried out at Schoen Clinic Newbridge, an adolescent inpatient eating disorders hospital in Birmingham. The patients involved in the group were offered support from occupational therapists, dieticians, nurses, psychologists and psychiatrists as part of their package of care from the inpatient hospital. Treatment included teaching practical skills including; support with food shopping and preparation, others were psychological addressing body image, self-esteem or family therapy. Other groups were psycho-educational. Medication was rarely used and always briefly, details have not been included in this research project. All treatments took place around the in-house school teaching programme which maintained the children’s education.

### Participants

Participants were female inpatients aged 11–18 who fulfilled DSM-V criteria for anorexia nervosa, no socio-demographic information was gathered. They were receiving treatment as an inpatient because local psychiatrists with no connection with the hospital deemed them too ill to receive treatment in the community.

Exclusion criteria for the study included moderate or severe learning disability, comorbid psychosis or other severe psychiatric condition or no English language ability. Inclusion and exclusion criteria were assessed using clinical documentation or through discussions with the multi-disciplinary team, responsible for the patient. No patients had a formal secondary diagnosis. A sample size of at least 12 was considered acceptable to generate exploratory findings as a pilot study. Informed consent was gained from all patients and their parents/carers. The intervention was authorised and reviewed by the Schoen Clinic Newbridge Research and Ethics Committee.

### Procedure

Young people were made aware of the group during their multidisciplinary meeting. Parents/carers were informed of a brief overview of the project and made aware that they would receive information and documentation via email. Young people were approached by an assistant psychologist at the same time. Both the patient and their parents/carers were told about the group in detail. Written details were provided. Informed consent was gained from both the patient and their parents/carers. Those who met the inclusion criteria were invited to participate. Participants were not compensated for taking part.

### Design

The I-ERP group is a four-session manualised programme developed for adolescents. The first author devised a Workbook and Therapists Manual (see Supplementary Material for copies). These were based on the I-ERP manual for adults created by Professor Cheri Levinson and adapted with her permission.

The first author, in trial testing sessions with patients concluded the treatment would need to be brief if the children’s attention is to be held. AN in adolescence can be less profound than in adults and long therapy sessions, for many children, may not be needed.

The authors decided that as school children are familiar with the concept of homework, using smart devices and being in classes, these would form a major feature of the new treatment. The treatment was delivered in group format with self-directed work between sessions. We introduced audio recordings (see below) to improve accessibility for the adolescents and reduced the number of sessions from 10 to 4. In addition to the Therapist Guide, the first author created Facilitator Notes which details how the group, session by session, was conducted. (These, together with the Patient Workbook, can be found in Supplementary Material.)

Session 0 was completed in a 1:1 setting, in which the young person meets with the therapist, to develop their own imaginal exposure script. Details of the procedure can be found in the Facilitator’s Notes. The script was written and recorded on an audio device. Each patient was instructed to read their script and listen to the recording each day as homework. Session 0 identified the patient’s core fears and the content of the subsequent four therapy sessions was established. All patients were given a Workbook (see Supplementary Material for the Workbook). The script was written by the patient with guidance using several prompts and using an adapted screening tool included in the original manual.

The sessions focused on doing exposure within the sessions whilst recording subjective units of distress (SUDS) (see figure). Patients completed a SUDS graph, in the I-ERP Workbook at the end of each session. This recorded changes within and between sessions. The group therapist emphasised the importance of out-of-session exposure, so patients felt responsible for behavioural change. The therapist's role was to offer information, psychoeducation, and facilitate exposure within the sessions, by giving support when the script was read aloud and ensuring the subjects marked subjective units of distress (SUDS). Sessions were 60 min and occurred weekly.

Patients were encouraged to feel their anxiety when listening to their audio recording of their scripts and encouraged to refrain from engaging in safety behaviours. Patients were strongly encouraged to listen to their script daily outside of sessions and record their SUDS which was reviewed in each session. In the final session, patients were asked to share their reflections and qualitative feedback about their experience of the group.

### Measures

#### Eating Disorder Examination-Questionnaire (EDE-Q; [13])

The EDE-Q is a self-report questionnaire consisting of 28 items. The questionnaire assesses the frequency and severity of behaviours that are associated with AN. The questionnaire has four subscales which assess restraint, eating concern, shape concern, and weight concern. The questionnaire generates an overall global score where a higher score is indicative of more problematic eating concerns. The EDE-Q shows good internal consistency and re-test reliability [38].

#### The Revised Child Anxiety and Depression Scale (RCADS) [6]

The RCADS is a 47-item, youth self-report questionnaire consisting of four subscales to assess separation anxiety disorder, social phobia, generalised anxiety disorder, panic disorder, obsessive–compulsive disorder, and major depressive disorder (low mood), it also creates a total anxiety score and total internalising score. Higher scores on the scale indicate increased severity of behaviours. The RCADS shows robust internal consistency reliability and good test–retest reliability [35].

#### Fear of Food Measure (FOFM; [26])

The FOFM is a 25-item self-report questionnaire that measures fears related to eating containing three subscales; anxiety about eating, feared concern, and food anxiety behaviours. FOFM has been demonstrated to show sensitivity to changes in fear during exposure, internal consistency ranged from good to excellent [26].

#### Subjective units of distress (SUDS; [44])

SUDS is a one-item 11-point Likert scale for subjective anxiety. SUDS measures anxiety, anger, distress and other internal experiences. The SUDS measure showed convergent validity with state anxiety, discriminant validity with trait anxiety and concurrent validity [22]. Evidence of predictive validity was also demonstrated [22].

### The feedback form

Qualitative feedback was collected following completion of the programme. It included open-ended questions asking the young people “What is your opinion of the group overall”, “What did you find most helpful”, “What did find least helpful”, “How acceptable did you find the group”, “What have you taken from the group”, “What have you taken away from the group”, “Are there any ways we could improve the group”.

### Statistical analysis

This study was a pilot study. The main aim of data analysis was to generate evidence by exploring the group's potential effectiveness to justify a full trial. Data were analysed using Excel and SPSS Statistics. The sample size was determined by opportunity sampling. Changes from pre (T1) to post-treatment (T2) were examined using paired *t*-tests. Missing data were excluded from the analysis. In addition, to evaluate clinical significance, Cohen’s D was calculated to provide effect sizes, with an effect size of 0.2 defined as small, 0.5 defined as medium and 0.8 defined as large [8]. No Bonferroni correction was completed due to conducting a pilot study.

## Results

Nineteen patients participated and none had a formal secondary diagnosis. All completed the four sessions across five separate groups. No patients dropped out of the group.

Means and standard deviation of eating disorder psychopathology (EDE-Q) are shown in Table 1. Anxiety and depression (RCAD’s) scores are shown in Table 2 and fear of food (FOFM) in Table 3. Scores were calculated before the group (T1) and after the group (T2). Mean scores decreased between T1 and T2 for all measures and subscales, indicating reduced eating behaviour psychopathology, anxiety and depression and suggests the group may be a helpful adjuvant to treatment.

**Table 1:** Overview of the group content

Session number	Programme structure	
	Session outline	Homework
0	Assessment and psychoeducation Screening for ED fears Writing and recording script Record SUDS Complete pre-outcome measures	
1	Common responses to exposure Explore safety behaviours Listening to script Record SUDS Complete SUDS graph	Listen to audio recording of the script and record SUDS
2	Common responses to exposure Explore safety behaviours Listening to script Record SUDS Complete SUDS graph	Listen to audio recording of the script and record SUDS
3	Common responses to exposure Explore safety behaviours Listening to script Record SUDS Complete SUDS graph	Listen to audio recording of the script and record SUDS
4	Common responses to exposure Explore safety behaviours Listening to script Record SUDS Complete SUDS graph What I Learned in Exposure Therapy or What I know About Anxiety Now Complete post-outcome measures

**Table 2:** Comparison of means for eating disorder psychopathology outcomes at T1 and T2

	T1		T2	
	Mean	SD	Mean	SD
EDEQ—weight concern	4.3273	1.73729	3.4909	2.06759
EDEQ—shape concern	4.9341	1.73148	3.4659	2.05713
EDEQ—eating concern	2.8727	1.50538	2.8545	2.18008
EDEQ—dietary restraint	3.0364	1.88853	2.0727	1.78723
EDEQ—global score	3.9056	1.59343	2.9313	1.78956

**Table 3:** Comparison of means for anxiety and depression outcomes at T1 and T2

	T1		T2	
	Mean	SD	Mean	SD
RCAD—separation anxiety	55.8182	25.81402	36.5455	31.20373
RCAD—social phobia	57.5455	20.61729	47.9091	24.84131
RCAD—general anxiety	48.1818	21.33456	38.0000	23.24220
RCAD—panic disorder	56.6364	23.23047	50.9091	30.59560
RCAD—OCD RCAD	48.6364	25.06900	47.3636	29.53057
RCAD—anxiety	66.3636	16.26821	55.3636	27.22599
RCAD—total	69.8182	21.18404	59.5455	29.17658

### Changes in eating disorder psychopathology

Mean and mean change in eating disorder psychopathology (EDE-Q Total) from pre- to post- groups were calculated (Table 2). Paired sample *t*-tests were used to compare the means (Table 5). This analysis demonstrated that there is not a significant reduction in eating disorder psychopathology.

### Changes in anxiety and depression

Mean and mean change in anxiety and depression (RCADS) from pre- to post-groups were calculated (Table 3). Paired sample *t*-tests were used to compare the means (Table 5). There was a significant reduction in anxiety *t*(10) = 2.657, *p* = 0.024, separation anxiety *t*(10) = 2.745, *p* = 0.021, general anxiety *t*(10) = 2.772, *p* = 0.020, and social phobia *t*(10) = 2.977, *p* = 0.014.

### Changes in fear of food

Mean and mean change in fear of food (FOFM) from pre- to post-groups were calculated (Table 4). Paired sample *t*-tests were used to compare the means (Table 5). There was no significant difference between subscales as seen in Table 5.

**Table 4:** Comparison of means for fear of food outcomes at T1 and T2

	T1		T2	
	Mean	SD	Mean	SD
FOFM—AE	43.8333	18.04901	33.8333	23.24149
FOFM—FAB	31.5556	10.60791	23.5556	13.77599
FOFM—FC	42.1000	16.26482	36.6000	17.93321

**Table 5:** Paired T-test from pre- to post-treatment

Paired samples test										
		Paired differences					*t*	*df*	Significance	
		Mean	Std. deviation	Std. error mean	95% Confidence interval of the difference				One-sided *p*	Two-sided *p*
					Lower	Upper				
Pair 1	T1EDEDR–T2EDEDR	0.96364	1.74544	0.52627	− 0.20896	2.13624	1.831	10	0.049	0.097
Pair 2	T1EDEEC–T2EDEC	0.01818	1.44901	0.43689	− 0.95528	0.99164	0.042	10	0.484	0.968
Pair 3	T1EDEWC–T2EDEWC	0.83636	1.42216	0.42880	− 0.11906	1.79179	1.950	10	0.040	0.080
Pair 4	T1EDESC–T2EDESC	1.46818	1.71472	0.51701	0.31622	2.62015	2.840	10	0.009	0.018*
Pair 5	T1EDEGLOBAL–T2EDEGLOBAL	0.97431	1.01377	0.33792	0.19505	1.75356	2.883	8	0.010	0.020*
Pair 6	T1RCADS_SA–T2RCADS_SA	19.27273	23.28558	7.02087	3.62926	34.91619	2.745	10	0.010	0.021*
Pair 7	T1RCADS_GA–T2RCADS_GA	10.18182	12.18046	3.67255	1.99887	18.36476	2.772	10	0.010	0.020*
Pair 8	T1RCADS_PAN–T2RCADS_PAN	5.72727	14.38117	4.33609	− 3.93413	15.38868	1.321	10	0.108	0.216
Pair 9	T1RCADS_SP–T2RCADS_SP	9.63636	10.73567	3.23693	2.42404	16.84868	2.977	10	0.007	0.014*
Pair 10	T1RCADS_OC–T2RCADS_OC	1.27273	17.35564	5.23292	− 10.38695	12.93240	0.243	10	0.406	0.813
Pair 11	T1RCADS_DEPRESSION–T2RCADS_DEPRESSION	2.90909	13.61216	4.10422	− 6.23568	12.05386	0.709	10	0.247	0.495
Pair 12	T1CARDS_ANXIETY–T2RCADS_ANXIETY	11.00000	13.73317	4.14071	1.77393	20.22607	2.657	10	0.012	0.024*
Pair 13	T1RCADS_TOTALANXDEP–T2RCADS_TOTALANXDEP	10.27273	15.69771	4.73304	− 0.27314	20.81859	2.170	10	0.028	0.055
Pair 14	T1_FOFM_AE–T2_FOFM_AE	10.00000	20.04994	8.18535	− 11.04112	31.04112	1.222	5	0.138	0.276
Pair 15	T1_FOFM_FAB–T2_FOFM_FAB	8.00000	12.16553	4.05518	− 1.35125	17.35125	1.973	8	0.042	0.084
Pair 16	T1_FOFM_FC–T2_FOFM_FC	5.50000	16.09865	5.09084	− 6.01628	17.01628	1.080	9	0.154	0.308

*Shows a significant difference

### Patient feedback

Qualitative information was gathered from patients who completed the group. Comments were overwhelmingly positive and suggested that the group had been a learning experience. They included “The more I face something anxiety provoking, the easier it gets”. Patients described that the group felt “challenging and helpful” as they had been able to confront core fears in a safe environment and learn that they are able to tolerate their fears. Other comments included: “Things that make me feel anxious are not as bad they seem”, “Safety behaviours work in the short, but not the long term”, “If I stay in a situation that is uncomfortable long enough, eventually it will not be uncomfortable” and “Things that I am afraid of happening are likely not as bad as I think or are less likely to happen than I think”. Feedback also suggested the treatment was the right length of time and any more sessions would be “boring”, otherwise there were no negative reports.

## Discussion

This was a pilot study, and excessive claims will not be made, recognising the numbers of patients treated and lack of a control. However, the findings are of interest and beg further work. To the authors’ knowledge, this is the first study to develop and test an imaginal exposure response prevention group, manualised and adapted to appeal to adolescents with AN and which specifically targets the core fears associated with being a normal body weight and anxiety in AN.

The I-ERP group reported significantly reduced anxiety, particularly, shape concern, anxiety, separation anxiety, general anxiety and social phobia. The statistical significant association was shown despite being offered the therapy as an adjuvant to a fully comprehensive package of care within an inpatient hospital.

These findings suggest that imaginal exposure response techniques are potentially successful to target core phobic fears and anxiety in AN and to improve eating disorder psychopathology and behaviours. Qualitative feedback gathered from patients who completed the group indicated that the group was challenging and beneficial which is a consistent theme across exposure therapy. Furthermore, no patients dropped out of the group which indicated that the intervention was acceptable to users. Several patients described how their anxiety had reduced after the group, and they felt less phobic of normal body weight as they learnt that the more they face something anxiety provoking, the easier it gets and things that make them uncomfortable or anxious are usually not as bad they seem. It is hoped that participation in the group will assist patients when they face similar situations in reality [20].

We acknowledge our debt to Levison and her team. Our results are similar [27–29] though our therapy was briefer, designed for young adolescents, not adults, done in group format, not one-to-one, and offered in an inpatient hospital, not in the out-patient clinic or on line. Levison, Rapp and Riley [27] addressed fear of fat whilst our treatment concentrated on fear of normal weight. The similarity in response adds perhaps to face validity of the approach.

It should be noted that these findings are limited as there was not a control group, therefore it is not possible to confirm that the I-ERP group was solely responsible for the reduction in eating disorder psychopathology and anxiety. It is possible that measured improvements in the group may be due other factors of inpatient treatment. The next stage should be to include a randomised controlled trial, and testing the programme in other settings, particularly as part of day-care or in an out-patient clinic.

Future research may consider comparing the effectiveness of I-ERP group with other exposure interventions as guidelines from the National Institute for Health Care and Excellence [33] recommend a comparison of group versus individual psychological interventions for eating disorders. Based on the rationale of the framework, it was not surprising that greater improvements are seen for anxiety as exposure therapy has been used to improve symptoms of anxiety in other mental health conditions including OCD [23] and PTSD [15, 16, 45].

The results suggest that the group may target core fears to tackle anxiety in AN, and it is encouraged that further research into the use of I-ERP to treat AN is conducted. To that end the Therapist Manual, Facilitator Notes and Patient Workbook are available here.

### What is already known on this subject?

There is no published evidence-based manualised imaginal exposure therapy for the treatment of anxiety and weight phobia in adolescent AN patients. Treatment designed for adults was shown to be effective. The authors devised and tested an imaginal exposure group therapy manual based on child-centred evidence.

### What does this study add?

This novel manualised group therapy has been specifically devised by the authors to treat AN in adolescent patients, when used as an adjuvant with other treatments. There is a significant association with reduced anxiety around weight, shape, social phobia, separation anxiety general anxiety and is acceptable by the patients.

### Limitations

This study was not a randomised control trial of I-ERP. Therefore, we were not able to compare its efficacy to ‘Treatment as Usual’ (TAU) or another control group. We were unable to conclude that I-ERP led to significant changes as patients received other support and treatment as part of their inpatient treatment. Furthermore, patients were unable to be followed up making it difficult to suggest that results are robust. In addition, the group was conducted in an inpatient setting so we are not able to ascertain that results would be similar in outpatient settings. Despite this, previous research [5] which used I-ERP to treat adults in a community-setting showed significant results so there is no clear reason that these results could not be replicated in outpatient settings. The research was conducted on a female population, due to the nature of the condition. Further research will be required to assess whether the treatment is of equal benefit to boys and girls. There was no suggestion that it would not be so. Finally, all adolescents who participated in the study had a primary diagnosis of AN; its efficacy in other eating disorders needs to be examined.

## Conclusion

The study finds that a specially designed, novel, manualised I-ERP group for adolescents with AN in a short format may be effective and acceptable to reduce weight and shape anxiety together with social and separation anxiety whilst being used as an adjuvant to inpatient treatment. However, this study does not include a control group meaning the authors cannot establish the groups effectiveness in isolation.

## Supplementary Information

Below is the link to the electronic supplementary material.

Supplementary Material 1

Supplementary Material 2

Supplementary Material 3

## Data Availability

No datasets were generated or analysed during the current study.
