# Supplementary material for: A pilot evaluation of the efficacy and acceptability of a novel imaginal exposure prevention (I-ERP) group programme to treat core weight, shape and social fears or phobias in adolescents with anorexia nervosa in an inpatient setting
Source: Eat Weight Disord. 2025 Feb 15;30(1):16. doi: 10.1007/s40519-025-01723-6 (PMC11829905; doi:10.1007/s40519-025-01723-6)
Supplement: Supplementary file 1 — Supplementary Material 1 [file 40519_2025_1723_MOESM1_ESM.docx]

**I-ERP Facilitator Notes**

**Kelsie Smith, Jessica Grant, Hubert Lacey**

**Session 0 - Assessment and Psycho education**

**Resources:**

ED fear handout, SUDS scale, pen.

**Aim:** Establish what the young person’s difficulties and goals are

**Session content:** This session introduces the young person to I-ERP and the theory of avoidance and habituation. The session then works on screening fears for the young person to work on.

***Introduction to I-ERP for adolescents (20 minutes)***

- What does I-ERP stand for? – imaginal exposure therapy
- What does this mean?
- I-ERP works on the principles on avoidance, exposure and habituation. The intervention will require you to expose yourself to your ‘core fears’ via your imagination.
- It can be hard to expose yourself to many eating disorder related fears, such as being abandoned. So we use something called imaginal exposure therapy. In imaginal exposure therapy we have you write about and imagine your fear. This type of exposure therapy has been shown to be just as effective as doing exposure therapy in real life.
- Avoidance - It’s natural and important to try to avoid things that make you anxious, that’s what anxiety is for. For example, it is natural to try and avoid bears, because they could hurt you.
- The problem with anxiety comes when, you have become anxious of something that can’t really hurt you. For example, food is not dangerous, but your body might react to it as though it is. That motivates you to avoid eating or to engage in unhealthy eating behaviours. If you avoid eating and avoid allowing yourself to experience any fears related to eating, you never get to see that your fears don’t really come true, or if they do come true, that they aren’t as bad as you thought they might be.
- Anxiety - When you do something anxiety provoking, anxiety usually peaks either toward the beginning of the event or before you begin (for example, it might be really nerve wrecking to give a public speech, but after you get going you become more comfortable with it). Most people’s anxiety goes down, at least a little bit, once they start. Alternatively, some people with eating disorders may notice that anxiety goes up again after a meal. This is common because the feeling of fullness can provoke anxiety all over again. However, in the same way that anxiety may go down at the beginning of a meal, anxiety does go back down again over time. Anxiety going up and then going back down again is part of a process called habituation. Habituation basically means “getting used to” something.
- Each time you face your fear, you are more likely to see your anxiety go down. Eventually your anxiety should reduce to the point that you can tolerate it.
- We measure anxiety using SUDS Scale. That stands for “Subjective Units of Distress’
- **Place a copy of the SUDS Scale in front of the participants.**
- The SUDS scale goes from 0 to 100. 0 means that you’re completely comfortable and relaxed. 25 means that you feel enough anxiety to get you going, but you don’t feel uncomfortable—the anxiety is actually helping you out. A 50 is moderate anxiety, somewhat bothersome. A 75 is bothering you enough that you’re considering leaving the situation entirely. A 100 is the worst anxiety you’ve ever experienced in your life. It can be any number between 0 and 100—those anchors are just there to give you an idea of what different ratings mean. Does all that make sense?
- **What would your SUDS rating be right now?”**
- **“What would your SUDS rating be when you are imagining (INSERT THEIR FEAR)?”**
- We will be asking you for your SUDS rating at various points during the imaginal exposure. When we ask you about your SUDS, don’t think too much about it. Just give a number quickly and move on to the next thing.

***Expectations of therapy (10 minutes)***

- **Prepare to feel anxious**. It is normal to feel uncomfortable when doing exposure therapy. In fact, if you become anxious it’s a sign that you are doing exposure correctly. Your job is to remain in the situation until your anxiety subsides on its own.
- **Don’t fight the anxiety or fear**. If you try to resist or fight the anxiety, you’ll only make things worse. Instead, let the anxious feelings just be there. Remember that they’re temporary. The worst thing that will happen is temporary distress.
- **Do not use safety behaviors** before, during, or after exposure. In order to work properly, exposure practices must be completed without coping or safety behaviors, seeking comfort or reassurance from others, distraction, or other strategies that make you feel safer or that prevent you from becoming anxious. Even very small or brief safety behaviors will spoil exposure. This means that you should not engage in behaviors like body checking or asking family members for reassurance. The point is to be anxious, so anything at all that might reduce anxiety will defeat the purpose of the exposure.
- **Keep track of your SUDS**. During imaginal exposure monitor how distressed you become using the SUDS scale from 0 (calm) to 100 (extremely anxious). Pay attention to whether your level of fearfulness changes as time goes by. We will be asking you periodically to record your SUDS.
- **A normal response to exposure therapy is to feel exhausted/tired/some depression** after completing an exposure session. Exposure is hard work and your body and mind will be tired out afterwards. If you experience these feelings, it means you are working hard and doing the exposure therapy exactly how you should be! Try and give yourself some self-care afterwards, you deserve it!

***Brief description of the group and structure (5 minutes)***

- 1 session per week (60 minutes)
- The group lasts 4 weeks
- Explain that the young person must read I-ERP script every day outside of session and rate their SUDS
- Reflection on homework at the beginning of each session
- There will be approximately 4 young people in each group

***Answer any question/worries the young person may have about starting the group (5 minutes)***

- Ask the young person what they would like to get from the I-ERP group (ensure these are realistic goals)
- Ask if they have any questions, concerns or worries about the group. Validate any concerns they may have
- Problem solve any worries they have e.g. fear of sharing in the group - Explain why the young people have been chosen for the group as they all have similar difficulties – referral from MDT may help them to feel more comfortable in opening up with their peers
- Discuss the importance of practice and ensure there is commitment
- Discuss importance of them making the choice to come to I-ERP (If there is a lack of motivation not appropriate)

**TASK:** Screen ED fears

**The SUDS Scale**


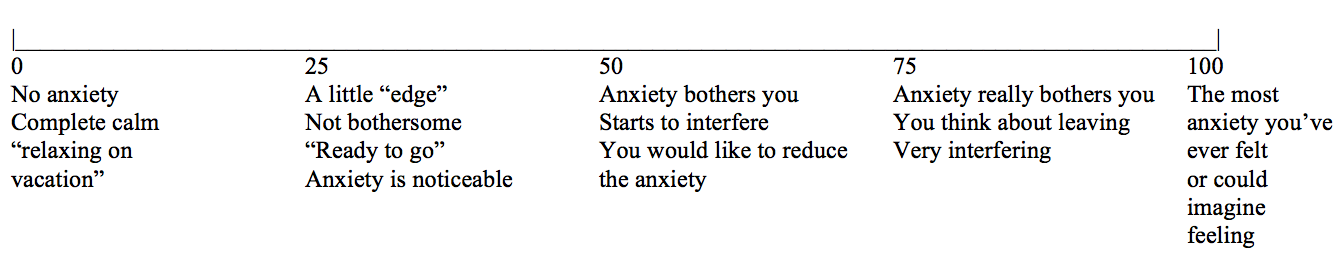


You can use any number between 0 and 100.

The anchors above are descriptions to give you a sense of the scale.

**SCREENING FOR ED FEARS**

***List out participant fears. For each fear listed, ask: “****On a scale of 1 to 10, with 1 being ‘no fear at all’ and 10 being ‘the worst fear possibly imagined,’ how would you rate this fear?”* ***For more general fears (e.g., rejection) confirm that fear is related to eating disorder. Read back the top three highest fears listed. Ask the participant which is their highest fear they would like to focus on.***

**NOTE: 1 = “No Fear At All”; 10 = “The Worst Fear Possibly Imagined”**

| Fear | Yes | No | Scale (1-10) | Check Top 3 Highest Fears |
| --- | --- | --- | --- | --- |
| Food | **☐** | **☐** |  | **☐** |
| Food with Unspecified Calories (AKA Not Knowing How Many Calories Are in the Meal) | **☐** | **☐** |  | **☐** |
| Foods Not Portioned Out | **☐** | **☐** |  | **☐** |
| Foods Containing a High Amount of Calories | **☐** | **☐** |  | **☐** |
| Specific Times of the Day (i.e. Meal Time) | **☐** | **☐** |  | **☐** |
| Extra Food (i.e. Ketchup, Mustard) | **☐** | **☐** |  | **☐** |
| Desserts or Sweets | **☐** | **☐** |  | **☐** |
| Processed Foods | **☐** | **☐** |  | **☐** |
| Specific Foods (i.e. Red meat, Sugar--- Ask participant if they maintain a specific diet) | **☐** | **☐** |  | **☐** |
| Eat More Food Than You Can Burn Via Exercise | **☐** | **☐** |  | **☐** |
| Not Exercising | **☐** | **☐** |  | **☐** |
| Weighing More Than a Specific Number | **☐** | **☐** |  | **☐** |
| Tolerating Your Size/ Not Accepting Your Body | **☐** | **☐** |  | **☐** |
| Fatty Foods | **☐** | **☐** |  | **☐** |
| Gaining Weight | **☐** | **☐** |  | **☐** |
| Rejection | **☐** | **☐** |  | **☐** |
| Abandonment | **☐** | **☐** |  | **☐** |
| Judgment | **☐** | **☐** |  | **☐** |
| Laziness | **☐** | **☐** |  | **☐** |
| Making Mistakes | **☐** | **☐** |  | **☐** |
| Feeling Physically Uncomfortable | **☐** | **☐** |  | **☐** |
| Feeling Emotionally Uncomfortable | **☐** | **☐** |  | **☐** |
| Losing Control | **☐** | **☐** |  | **☐** |
| Being Compared | **☐** | **☐** |  | **☐** |
| Judgment on Appearance | **☐** | **☐** |  | **☐** |
| Judgment on Social Media | **☐** | **☐** |  | **☐** |
| Failing to Meet Expectations (i.e Familial Expectations of What Your Size Should Be) | **☐** | **☐** |  | **☐** |
| Judgment of Self | **☐** | **☐** |  | **☐** |
| Failing to Meet Societal Expectations | **☐** | **☐** |  | **☐** |
| Embarrassment About Appearance | **☐** | **☐** |  | **☐** |
| Embarrassment/Discomfort | **☐** | **☐** |  | **☐** |
| Embarrassed about your emotions | **☐** | **☐** |  | **☐** |

| **“Are there any other fears that you have that I have not mentioned?” If YES, Please describe them below:** | | | |
| --- | --- | --- | --- |
|  | | | |
| **TOP 3 Fears:**  **(Please write in each fear at the top)** |  |  |  |
| **What do they fear specifically? (I.E. Types of Food feared)** |  |  |  |

**IMAGINAL EXPOSURE TRAINING: Participant Requirements**



|  | Yes | No |
| --- | --- | --- |
| Have you ever been in exposure therapy before? | ☐ | ☐ |
| -- if YES, What was your experience like? (write description below) | | |
|  | | |
| What would your SUDS rating be right now? | ________ | |
| What would your SUDS rating be when you are imagining (INSERT PARTICIPANT’S FEAR)? | ________ | |
| (For Consenter ONLY) Does the Participant’s SUDS rating make sense? | ☐ | ☐ |
| -- if NO, please explain (write description below) | | |
|  | | |
| Did patient explain that he/she felt good about how to use the SUDS rating system? | ☐ | ☐ |
| In the space below, please record any other pertinent information regarding the participant’s concerns/thoughts/etc.: | | |
|  | | |

**I-ERP Information Sheet**

**Introduction to I-ERP**

- What does I-ERP stand for? – imaginal exposure therapy
- What does this mean?
- I-ERP works on the principles on avoidance, exposure and habituation. The intervention will require exposure to ‘core fears’ via imagination.
- Exposure is difficult for eating disorder related fears, such as being abandoned. So, we use something called imaginal exposure therapy. In imaginal exposure therapy we have the young person write about and imagine their fear. This type of exposure therapy has been shown to be just as effective as doing exposure therapy in real life.
- Avoidance - It’s natural and important to try to avoid things that cause anxiety, that’s what anxiety is for. For example, it is natural to try and avoid bears, because they could hurt you.
- The problem with anxiety comes when, the young person has become anxious of something that can’t really hurt them. For example, food is not dangerous, but the body might react to it as though it is. That motivates people to avoid eating or to engage in unhealthy eating behaviours. If they avoid eating and avoid allowing themselves to experience any fears related to eating, young people never get to see that their fears don’t really come true, or if they do come true, that they aren’t as bad as they thought they might be.
- Anxiety - When you do something anxiety provoking, anxiety usually peaks either toward the beginning of the event or before you begin (for example, it might be really nerve wrecking to give a public speech, but after you get going you become more comfortable with it). Most people’s anxiety goes down, at least a little bit, once they start. Alternatively, some people with eating disorders may notice that anxiety goes up again after a meal. This is common because the feeling of fullness can provoke anxiety all over again. However, in the same way that anxiety may go down at the beginning of a meal, anxiety does go back down again over time. Anxiety going up and then going back down again is part of a process called habituation. Habituation basically means “getting used to” something.
- Each time they young person faces their fear, they are more likely to see their anxiety go down. Eventually their anxiety should reduce to the point that they are able tolerate it.

**Intervention Structure**

- The intervention consists of 4 live sessions.
- There will be one session per week which will last approximately 60 minutes.
- There is an expectation that the young person will complete exposure homework daily and record this until the next session.

**What to expect?**

- The young person has been informed to; prepare to feel anxious, the importance of not fighting the anxiety or fear and not utilising safety behaviours.
- The young person will also be engaged in individual therapy so there is wrap around support and this intervention can be discussed in their individual sessions.

**I-ERP Script**

**Ask: “What is your SUDS right now?” _____________ (Ask every 5-8 minutes)**

**Imaginal Script Session 3 (Page 1/4)**

“First, recall a specific fear you have at this time in your life. Start at the beginning—the context is currently being an inpatient at Newbridge.”

___________________________________________________________________________

___________________________________________________________________________

___________________________________________________________________________

___________________________________________________________________________

___________________________________________________________________________

___________________________________________________________________________

___________________________________________________________________________

___________________________________________________________________________

**SUDS: ______** “What is leading up to this specific fear happening? Give as many details as possible.”

“What do you feel in your body right now? What emotions are you feeling right now? What thoughts are going through your head right now?”
________________________________________________________________________

___________________________________________________________________________

___________________________________________________________________________

___________________________________________________________________________

___________________________________________________________________________

___________________________________________________________________________

___________________________________________________________________________

___________________________________________________________________________

___________________________________________________________________________

___________________________________________________________________________

___________________________________________________________________________

___________________________________________________________________________

___________________________________________________________________________

“Imagine you are a normal weight and due to be discharged, how do you feel when your family see you; how do you feel when others see you for the first time?”

“What do you feel in your body right now? What emotions are you feeling right now? What thoughts are going through your head right now?”

**(Page 2/4)**

___________________________________________________________________________

___________________________________________________________________________

___________________________________________________________________________

___________________________________________________________________________

___________________________________________________________________________

___________________________________________________________________________

___________________________________________________________________________

___________________________________________________________________________

“Imagine you are at normal weight, how to you feel when you walk into school, into your class on the first occasion; into the school dining room”

“What do you feel in your body right now? What emotions are you feeling right now? What thoughts are going through your head right now?”

___________________________________________________________________________

___________________________________________________________________________

___________________________________________________________________________

___________________________________________________________________________

___________________________________________________________________________

___________________________________________________________________________

___________________________________________________________________________

______________________________________________________________________________________________________________________________________________________

___________________________________________________________________________

___________________________________________________________________________

**SUDS______** “Imagine you are at normal weight; how would you feel in a swimsuit on the beach?”

___________________________________________________________________________

___________________________________________________________________________

___________________________________________________________________________

___________________________________________________________________________

___________________________________________________________________________

___________________________________________________________________________

___________________________________________________________________________

___________________________________________________________________________

___________________________________________________________________________

___________________________________________________________________________

___________________________________________________________________________

“You are at a normal weight and a close school friend invites you to her birthday party - the first you have been to - and there are both boys and girls there, some you know and some you don’t. There is food and the cake is offered around”

“What do you feel in your body right now? What emotions are you feeling right now? What thoughts are going through your head right now?”

___________________________________________________________________________

___________________________________________________________________________

___________________________________________________________________________

___________________________________________________________________________

___________________________________________________________________________

___________________________________________________________________________

___________________________________________________________________________

___________________________________________________________________________

___________________________________________________________________________

___________________________________________________________________________

___________________________________________________________________________

___________________________________________________________________________

______________________________________________________________________________________________________________________________________________________

**SUDS______** “Imagine you are at normal weight; you are going clothes shopping for the first time; you try on an item and it doesn’t fit correctly.”

“What do you feel in your body right now? What emotions are you feeling right now? What thoughts are going through your head right now?”

___________________________________________________________________________

___________________________________________________________________________

___________________________________________________________________________

___________________________________________________________________________

___________________________________________________________________________

___________________________________________________________________________

___________________________________________________________________________

___________________________________________________________________________

___________________________________________________________________________

___________________________________________________________________________

**SUDS______** “Now imagine your fear is coming to an end. What is happening? How does it end?”

___________________________________________________________________________

___________________________________________________________________________

___________________________________________________________________________

___________________________________________________________________________

___________________________________________________________________________

___________________________________________________________________________

___________________________________________________________________________

___________________________________________________________________________

___________________________________________________________________________

___________________________________________________________________________

___________________________________________________________________________

___________________________________________________________________________

___________________________________________________________________________

___________________________________________________________________________

**End SUDS: _______**

**SUDS SHEET**

**Date: __________**

**SUDS 1: ________**

**SUDS 2: ________**

**SUDS 3: ________**

**SUDS 4: ________**

**SUDS 5: ________**

**SUDS 6: ________**

**SUDS 7: ________**

**SUDS 8: ________**

**SUDS 9: ________**

**SUDS 10: ________**

**I-ERP Facilitator Notes**

**Kelsie Smith, Jessica Grant, Hubert Lacey**

**Session One**

**Resources:**

Handout, pen, paper, graphs

**Learning outcomes**

By the end of session one you should know more about:

- Imaginal Exposure Response Prevention
- Safety Behaviors

**INTRODUCTION TO GROUP THERAPY**

Introduce names and engage in an open discussion about experiences of previous groups attended. *“Does everyone know each other?”,* “*Has anyone attended group therapy before?”*

What groups look like? What to expect from groups?

- Talking about silence, talking about sharing and how scary that can be
- Group dynamics – there may be people you don’t know or feel less comfortable – importance of group boundaries

**SETTING GROUP BOUNDARIES (5 mins)**

Highlight that group rules / boundaries are set for the benefit of the young people, not for us. The aim of these rules is to make the group a safe and comfortable environment for the young people to speak openly about their opinions, experiences and reflections if they want to. Ask each young person to set at least one rule.

Write these on a large piece of paper which can be stuck to the wall for each session and ask the young people to also note them down in their workbooks – both are for future reference.

Some examples include:

- Listening to others
- Confidentiality
- Participating
- Being respectful
- No exercising (including leg shaking)
- Being prepared for sessions (toilet etc.)

If some of these aren’t on the young people’s lists, add them to the bottom.

Then introduce session one and read out the learning outcomes. Session one will look at the different types of eating disorders

**INTRODUCTION TO I-ERP (5 minutes)**

- Explain that everyone should have an overview of I-ERP from the assessment.
- Re-cap avoidance, habituation and exposure if necessary.
- We are going to be working as a group to expose young people to their ‘core fears’ through their imagination.
- We spoke about ‘safety behaviors’ at the assessment but we are going to speak about them in more detail today.

**SAFETY BEHAVIOURS (15 minutes)**

**DISCUSSION:** What are safety behaviours?

Safety behaviours are actions carried out with the to prevent experiencing a feared ‘catastrophe’. In the short-term they often give a sense of relief, but in the long-term they are unhelpful because they prevent us disproving the feared beliefs which maintain anxiety.

What is a catastrophe? - We expect disaster to strike, no matter what. This is also referred to as “magnifying or minimizing.” We hear about a problem and use *what if* questions (e.g., “What if tragedy strikes?” “What if it happens to me?”).

- Physical threat - *“I’ll be killed”, “I’ll be hurt”*
- Psychological threat - *“I’ll go mad”*, *“I can’t cope”*
- Social threat - *“I’ll embarrass myself and never be able to show my face again”, “They will think I’m an idiot”*
- Eating disorder threat – *“I’ll gain weight and never lose it.”*
- **DISCUSS:** What is your feared catastrophe?

**DISCUSS:** What types of safety behaviours are there?

There are four types of safety behaviours:

1. *Avoidance* - e.g. not going to a feared situation
2. *Escape* - e.g. leaving a feared situation
3. *Eating disorder safety behaviours* - exercising, counting calories, body checking, purging
4. *Subtle avoidance*, which can include things we do in our minds - e.g.
   - distraction - counting in my head during a panic to stop myself from going mad
   - calming my breathing - otherwise I’ll be overwhelmed by my fear and lose control
   - averting my eyes - in case someone picks on me and I’m humiliated
   - **DISCUSS:** What safety behaviour do you use?

**DISCUSS**: What are the effects of safety behaviours?

• Short term: In the short-term safety behaviours lead to a reduction in anxiety. Any form of escape or avoidance is often accompanied by a powerful feeling of relief. Relief is powerful negative reinforcer, and once an individual has learned that a safety behaviour leads to relief they are likely to use it again.

• Long term: In the longer term, safety behaviours act to maintain anxiety by preventing the disconfirmation of unhelpful beliefs. For example, if someone has the belief “dogs will attack me and bite my face” and avoids dogs, they don’t get the opportunity to learn that most dogs are friendly, or fail to learn the diﬀerence between friendly and unfriendly dogs.

• Unintended consequences: Safety behaviours often have unintended consequences which can reinforce the original belief, make the anxiety worse, or lead to other problems.

**DISCUSS:** What is the difference between a safety behaviour and adaptive behaviour?

- Just by looking at the behaviour itself, there is no way of telling the difference between an unhelpful safety behaviour and a helpful adaptive behaviour. It is the *intention* which matters - safety behaviours are those which are *intended* to avert a feared catastrophe.
- For example, if the strategy of distraction is used to cope with a painful experience (e.g. a visit to the dentist) we say it is helpful. However, if distraction is used with the *intention* of preventing a catastrophe (e.g. to avoid a feeling of panic that I fear will make me go mad) then it can be viewed as an unhelpful safety behaviour.

**TASK:** Read through scenario one on the table below

| **Early Experience** | **Belief** | **Safety Behaviour** | **Short Term Consequence** | **Long Term Consequence** | **Unintended Consequence** |
| --- | --- | --- | --- | --- | --- |
| Made fun of for appearance and being exposed to a thin-ideal beauty standard in the media | If I gain weight, I will never be able to lose it, no one will like me, I will be rejected and abandoned | Counting calories (restricting) and other behaviours (over -exercise) | Reduction in anxiety because I am in control of weight gain | No opportunity to learn that I won’t be abandoned or rejected if I lose weight, won’t learn that I don’t need to restrict and engage in compensatory behaviours to not gain weight | Difficulty enjoying eating food and relying on compensatory behaviours which impacts physical health and leads to disordered eating |
| Physically assaulted by a group of men walking home after I looked at them | People are dangerous, if I make eye contact with others, I will be attacked | Avert my eyes to avoid contact | Reduction in anxiety because I am not afraid that I will be attacked my others if I’m not looking at them | No opportunity to learn that I won’t be attacked if I make eye contact with others. | Difficulty sustaining and forming relationships with others because I will not look at people which may make others uncomfortable |
| Making a mistake on a presentation in school | If I make a mistake everything will be horrible | Spend all my time working on my presentation | Reduction in anxiety as I will feel less likely to make a mistake if I have spent all my time perfecting the work | No opportunity to learn that I do not need to spend an excessive amount of time on a project to do it to a good standard | Not spending time with friends and family or doing things I enjoy due to being occupied with perfecting school work |
| Parents getting divorced | People leave people who they love, relationships don’t last | Withdrawing from relationship due to scared of being rejected | Reduction in anxiety because I am not close enough to anyone for them to leave me and hurt me | No opportunity to learn that not all relationships fail and that I will not be rejected and left alone | No relationships with other people which leads to feeling lonely and having low self-esteem |

Any questions on anything we have covered today?

**Exposure (35 minutes)**

**TASK:** Patients to listen to their exposure scripts independently for 30 minutes and facilitator to ask patients to make a note of their SUDS at 5-minute intervals.

**DISCUSS:** Any reflections on exposure this week?

**TASK:** Complete ‘after’ SUD’s a couple of minutes after listening to their script and patients to complete the graph.

**Homework**

- Read exposure script to yourself every day in the morning until the next session. Complete the SUDS sheet and reflect next week.

**I-ERP Facilitator Notes**

**Kelsie Smith, Jessica Grant, Hubert Lacey​​**

**Session Two**

**Resources:**

Handout, pen, paper

**Learning outcomes**

By the end of session one you should know more about:

- Common responses to exposure
- Safety behaviors
- Avoidance hierarchy
- Exposure

**Homework practice review (5 minutes)**

Read the exposure script to yourself every day in the morning and record SUDS.

**If the patients don’t do the homework:**

- Ask when you left the last session did you understand the homework?
- If forgotten to complete – prolonged exposure is a vital part of this intervention and will not be as effective if not completed. What can we do about this? Write a note or enter into calendars?
- When you left the group were you motivated to practice the skill? – if it was a problem of motivation try to gain commitment to practice explore this in individual sessions or communicate difficulties with the facilitator
- Reflection – How did it feel to do repeated exposure, has there been any changes with the SUDS throughout exposure sessions?

**Common Responses to Exposure Therapy (5 minutes)**

**DISCUSS:** Exposure therapy is hard work and your body and mind may be tired out afterwards.

Normal responses to exposure therapy can include:

- Feeling tired
- Feeling exhausted
- Some depression

You may feel some of these things after completing an exposure session. If you experience these feelings, it means you are working hard and doing the exposure therapy exactly how you should be! Try and give yourself some self-care afterwards, you deserve it!

**Safety Behaviours (10 minutes)**

**DISCUSS:** Recap safety behaviours

**TASK:** Complete scenario two of the safety behaviours table (page 7)

| **Early Experience** | **Belief** | **Safety Behaviour** | **Short Term Consequence** | **Long Term Consequence** | **Unintended Consequence** |
| --- | --- | --- | --- | --- | --- |
| Made fun of for appearance and being exposed to a thin-ideal beauty standard in the media | If I gain weight, I will never be able to lose it, no one will like me, I will be rejected and abandoned | Counting calories (restricting) and other behaviours (over -exercise) | Reduction in anxiety because I am in control of weight gain | No opportunity to learn that I won’t be abandoned or rejected if I lose weight, won’t learn that I don’t need to restrict and engage in compensatory behaviours to not gain weight | Difficulty enjoying eating food and relying on compensatory behaviours which impacts physical health and leads to disordered eating |
| Physically assaulted by a group of men walking home after I looked at them | People are dangerous, if I make eye contact with others, I will be attacked | Avert my eyes to avoid contact | Reduction in anxiety because I am not afraid that I will be attacked my others if I’m not looking at them | No opportunity to learn that I won’t be attacked if I make eye contact with others. | Difficulty sustaining and forming relationships with others because I will not look at people which may make others uncomfortable |
| Making a mistake on a presentation in school | If I make a mistake everything will be horrible | Spend all my time working on my presentation | Reduction in anxiety as I will feel less likely to make a mistake if I have spent all my time perfecting the work | No opportunity to learn that I do not need to spend an excessive amount of time on a project to do it to a good standard | Not spending time with friends and family or doing things I enjoy due to being occupied with perfecting school work |
| Parents getting divorced | People leave people who they love, relationships don’t last | Withdrawing from relationship due to scared of being rejected | Reduction in anxiety because I am not close enough to anyone for them to leave me and hurt me | No opportunity to learn that not all relationships fail and that I will not be rejected and left alone | No relationships with other people which leads to feeling lonely and having low self-esteem |

**Avoidance Hierarchy (5 minutes)**

**TASK:** Construct a ladder of places or situations that you avoid. At the top of the ladder put those which make you most anxious. At the bottom of the ladder put places or situations you avoid, but which don’t bother you as much. In the middle of the ladder put ones that are ‘in-between’. Give each item a rating from 0-100% according to how anxious you would feel if you had to be in that situation. Overcome your anxiety by approaching these situations, starting from the bottom of the ladder.

**Exposure (35 minutes)**

**TASK:** Patients to listen to their exposure scripts independently for 30 minutes and facilitator to ask patients to make a note of their SUDS at 5-minute intervals.

**DISCUSS:** Any reflections on exposure this week?

**TASK:** Complete ‘after’ SUD’s a couple of minutes after listening to their script and patients to complete the graph.

**Homework**

- Read exposure script to yourself every day in the morning until the next session. Complete the SUDS sheet to reflect next week.

**I-ERP Facilitator Notes**

**Session Three**

**Resources:**

Handout, pen, paper

**Learning outcomes**

By the end of session one you should know more about:

- Common responses to exposure
- Safety Behaviours
- Exposure

**Homework practice review (5 minutes)**

Read the exposure script to yourself every day in the morning and record SUDS.

**If the patients don’t do the homework:**

- Ask when you left the last session did you understand the homework?
- If forgotten to complete – prolonged exposure is a vital part of this intervention and will not be as effective if not completed. What can we do about this? Write a note or enter into calendars?
- When you left the group were you motivated to practice the skill? – if it was a problem of motivation try to gain commitment to practice explore this in individual sessions or communicate difficulties with the facilitator
- Reflection – How did it feel to do repeated exposure, has there been any changes with the SUDS throughout exposure sessions?

**Common Responses to Exposure Therapy**

**DISCUSSION:** You may feel some of these things after completing an exposure session. If you experience these feelings, it means you are working hard and doing the exposure therapy exactly how you should be! Try and give yourself some self-care afterwards, you deserve it! Normal responses include;

- Feeling tired
- Feeling exhausted
- Some depression

**Safety Behaviours (10 minutes)**

DISCUSS: Recap safety behaviours

TASK: Complete scenario three of the safety behaviours table (page 7)

| **Early Experience** | **Belief** | **Safety Behaviour** | **Short Term Consequence** | **Long Term Consequence** | **Unintended Consequence** |
| --- | --- | --- | --- | --- | --- |
| Made fun of for appearance and being exposed to a thin-ideal beauty standard in the media | If I gain weight, I will never be able to lose it, no one will like me, I will be rejected and abandoned | Counting calories (restricting) and other behaviours (over -exercise) | Reduction in anxiety because I am in control of weight gain | No opportunity to learn that I won’t be abandoned or rejected if I lose weight, won’t learn that I don’t need to restrict and engage in compensatory behaviours to not gain weight | Difficulty enjoying eating food and relying on compensatory behaviours which impacts physical health and leads to disordered eating |
| Physically assaulted by a group of men walking home after I looked at them | People are dangerous, if I make eye contact with others, I will be attacked | Avert my eyes to avoid contact | Reduction in anxiety because I am not afraid that I will be attacked my others if I’m not looking at them | No opportunity to learn that I won’t be attacked if I make eye contact with others. | Difficulty sustaining and forming relationships with others because I will not look at people which may make others uncomfortable |
| Making a mistake on a presentation in school | If I make a mistake everything will be horrible | Spend all my time working on my presentation | Reduction in anxiety as I will feel less likely to make a mistake if I have spent all my time perfecting the work | No opportunity to learn that I do not need to spend an excessive amount of time on a project to do it to a good standard | Not spending time with friends and family or doing things I enjoy due to being occupied with perfecting school work |
| Parents getting divorced | People leave people who they love, relationships don’t last | Withdrawing from relationship due to scared of being rejected | Reduction in anxiety because I am not close enough to anyone for them to leave me and hurt me | No opportunity to learn that not all relationships fail and that I will not be rejected and left alone | No relationships with other people which leads to feeling lonely and having low self-esteem |

**Avoidance Hierarchy (10 minutes)**

**DISCUSS:** Revisit the avoidance hierarchy and discuss experiences completing exposure to these scarious, encourage patients to work their way up the ladder.

**Exposure (35 minutes)**

**TASK:** Patients to listen to their exposure scripts independently for 30 minutes and facilitator to ask patients to make a note of their SUDS at 5-minute intervals.

**DISCUSS:** Any reflections on exposure this week?

**TASK:** Complete ‘after’ SUD’s a couple of minutes after listening to their script and patients to complete the graph.

**Homework**

- Read exposure script to yourself every day in the morning until the next session. Complete the SUDS sheet to reflect next week.

**I-ERP Facilitator Notes**

**Kelsie Smith, Jessica Grant, Hubert Lacey**

**Session Four**

**Resources:**

Handout, pen, paper

**Learning outcomes**

By the end of session one you should know more about:

- Common responses to exposure
- Safety Behaviours
- Exposure
- What it was like to do I-ERP

**Homework practice review (5 minutes)**

Read the exposure script to yourself every day in the morning and record SUDS.

**If the patients don’t do the homework:**

- Ask when you left the last session did you understand the homework?
- If forgotten to complete – prolonged exposure is a vital part of this intervention and will not be as effective if not completed. What can we do about this? Write a note or enter into calendars?
- When you left the group were you motivated to practice the skill? – if it was a problem of motivation try to gain commitment to practice explore this in individual sessions or communicate difficulties with the facilitator
- Reflection – How did it feel to do repeated exposure, has there been any changes with the SUDS throughout exposure sessions?

**Common Responses to Exposure Therapy (5 minutes)**

**DISCUSSION:** You may feel some of these things after completing an exposure session. If you experience these feelings, it means you are working hard and doing the exposure therapy exactly how you should be! Try and give yourself some self-care afterwards, you deserve it! Normal responses include;

- Feeling tired
- Feeling exhausted
- Some depression

**Safety Behaviours (10 minutes)**

DISCUSS: Recap safety behaviours

TASK: Complete scenario four of the safety behaviours table (page 7)

| **Early Experience** | **Belief** | **Safety Behaviour** | **Short Term Consequence** | **Long Term Consequence** | **Unintended Consequence** |
| --- | --- | --- | --- | --- | --- |
| Made fun of for appearance and being exposed to a thin-ideal beauty standard in the media | If I gain weight, I will never be able to lose it, no one will like me, I will be rejected and abandoned | Counting calories (restricting) and other behaviours (over -exercise) | Reduction in anxiety because I am in control of weight gain | No opportunity to learn that I won’t be abandoned or rejected if I lose weight, won’t learn that I don’t need to restrict and engage in compensatory behaviours to not gain weight | Difficulty enjoying eating food and relying on compensatory behaviours which impacts physical health and leads to disordered eating |
| Physically assaulted by a group of men walking home after I looked at them | People are dangerous, if I make eye contact with others, I will be attacked | Avert my eyes to avoid contact | Reduction in anxiety because I am not afraid that I will be attacked my others if I’m not looking at them | No opportunity to learn that I won’t be attacked if I make eye contact with others. | Difficulty sustaining and forming relationships with others because I will not look at people which may make others uncomfortable |
| Making a mistake on a presentation in school | If I make a mistake everything will be horrible | Spend all my time working on my presentation | Reduction in anxiety as I will feel less likely to make a mistake if I have spent all my time perfecting the work | No opportunity to learn that I do not need to spend an excessive amount of time on a project to do it to a good standard | Not spending time with friends and family or doing things I enjoy due to being occupied with perfecting school work |
| Parents getting divorced | People leave people who they love, relationships don’t last | Withdrawing from relationship due to scared of being rejected | Reduction in anxiety because I am not close enough to anyone for them to leave me and hurt me | No opportunity to learn that not all relationships fail and that I will not be rejected and left alone | No relationships with other people which leads to feeling lonely and having low self-esteem |

**Avoidance Hierarchy (5 minutes)**

**DISCUSS:** Revisit the avoidance hierarchy and discuss experiences completing exposure to these scarious, encourage patients to work their way up the ladder.

**Exposure (45 minutes)**

**TASK:** Patients to listen to their exposure scripts independently for 30 minutes and facilitator to ask patients to make a note of their SUDS at 5-minute intervals.

**DISCUSS:** Any reflections on exposure this week?

**TASK:** Complete ‘after’ SUD’s a couple of minutes after listening to their script and patients to complete the graph.

**TASK:** Complete what I learned in therapy worksheet

**What I Learned in Exposure Therapy or What I know About Anxiety Now**

- The more I face something anxiety provoking the easier it gets
- If I feel like avoiding something because it makes me uncomfortable, I should face it instead
- Things that make me uncomfortable/anxious are usually not as bad they seem
- I should let myself feel my emotions
- Safety behaviors work in the short, but not the long term
- If you stay in a situation that is uncomfortable long enough, eventually it will not be uncomfortable
- Things that I am afraid of happening are likely not as bad as I think or are less likely to happen than I think
- I can handle my anxiety and my eating disorder!
- Other things I have learned:

_____________________________________________________________________

_____________________________________________________________________

_____________________________________________________________________

_____________________________________________________________________

_____________________________________________________________________

**Are there any outstanding comments or questions?**

**Collect Exposure scripts**

I-ERP Feedback

1. What is your opinion of the group overall?

2.What did you find most helpful?

3. What did you find least helpful?

4. How acceptable did you find the intervention (e.g homework, length of time, content?)

5. What have you taken away from this group?

6. Are they any ways we could improve this group?
