# Supplementary material for: A pilot evaluation of the efficacy and acceptability of a novel imaginal exposure prevention (I-ERP) group programme to treat core weight, shape and social fears or phobias in adolescents with anorexia nervosa in an inpatient setting
Source: Eat Weight Disord. 2025 Feb 15;30(1):16. doi: 10.1007/s40519-025-01723-6 (PMC11829905; doi:10.1007/s40519-025-01723-6)
Supplement: Supplementary file 2 — Supplementary Material 2 [file 40519_2025_1723_MOESM2_ESM.pdf]

Imaginal Exposure Response Prevention Therapy (I-ERP) Workbook

NAME: ....

Kelsie Smith, Jessica Grant, Professor Hubert Lacey

Psycho education

“Avoidance is a very important part of anxiety. It’s natural to try to avoid things that make you anxious. Actually, that’s what anxiety is for. It’s for motivating you to avoid things in the world that could hurt you. For example, it is natural to try and avoid bears, because they could hurt you.

The problem with anxiety comes when, for some reason, you have become anxious of something that can’t really hurt you. For example, food is not dangerous, but your body might react to it as though it is. That motivates you to avoid eating or to engage in unhealthy eating behaviours. If you avoid eating and avoid allowing yourself to experience any fears related to eating, you never get to see that your fears don’t really come true, or if they do come true, that they aren’t as bad as you thought they might be. Does that make sense?

When you do something anxiety provoking, anxiety usually peaks either toward the beginning of the anxiety provoking event or before you begin (for example, it might be really nerving to give a public speech, but after you get going you become more comfortable with it). Most people’s anxiety goes down, at least a little bit, once they start. Alternatively, some people with eating disorders may notice that anxiety goes up again after a meal. This is common because the feeling of fullness can provoke anxiety all over again. However, in the same way that anxiety may go down at the beginning of a meal, anxiety does go back down again over time. Anxiety going up and then going back down again is part of a process called habituation. Habituation basically means “getting used to” something.

The good news is that each time you face your fear, you are more likely to see your anxiety go down. Even though right now it may just be the difference between feeling very, very anxious and feeling uncomfortably anxious, eventually your anxiety should reduce to the point that you can tolerate it.

What I’ve just been describing is what’s called exposure therapy. Exposure is the main ingredient in nearly all of the effective treatments for anxiety. Psychologists have tried to come up with other methods, but exposure has been shown, time and again, to be a key component of successful treatment.

However, it can be hard to expose yourself to many eating disorder related fears, such as being abandoned. So, we use something called imaginal exposure therapy. In imaginal exposure therapy we have you write about and imagine your fear. This type of exposure therapy has been shown to be just as effective as doing exposure therapy in real life.

So, what does this mean for you? This means that as you complete imaginal exposures (writing about and imagining your fear) you will become less and less anxious about your fear. This will help you gain control of your fears and any eating disorder behaviours you experience.

We measure anxiety using SUDS Scale. That stands for “Subjective Units of Distress’

The SUDS scale goes from 0 to 100. 0 means that you’re completely comfortable and relaxed. 25 means that you feel enough anxiety to get you going, but you don’t feel uncomfortable—the anxiety is actually helping you out. A 50 is moderate anxiety, somewhat

bothersome. A 75 is bothering you enough that you're considering leaving the situation entirely. A 100 is the worst anxiety you've ever experienced in your life. It can be any number between 0 and 100—those anchors are just there to give you an idea of what different ratings mean.

Expectations of Therapy

Prepare to feel anxious. It is normal to feel uncomfortable when doing exposure therapy. In fact, if you become anxious it's a sign that you are doing exposure correctly. Your job is to remain in the situation until your anxiety subsides on its own. Remind yourself, the more I feel anxious, the faster this will work!

Don't fight the anxiety or fear. If you try to resist or fight the anxiety, you'll only make things worse. Instead, let the anxious feelings just be there. Remember that they're temporary. The worst thing that will happen is temporary distress.

Do not use safety behaviors before, during, or after exposure. In order to work properly, exposure practices must be completed without coping or safety behaviors, seeking comfort or reassurance from others, distraction, medications, alcohol, or other strategies that make you feel safer or that prevent you from becoming anxious. Even very small or brief safety behaviors will spoil exposure. This means that you should not engage in behaviors like body checking or asking family members for reassurance. The point is to be anxious, so anything at all that might reduce anxiety will defeat the purpose of the exposure.

REMEMBER TO STAY IN THE PRESENT TENSE. When you are writing about or remembering your fear, make sure to stay in the present. Write about it as if it were happening to you right now. This will help the situation seem more real, and over time will give you more control over it.

Keep track of your SUDS. During imaginal exposure monitor how distressed you become using the SUDS scale from 0 (calm) to 100 (extremely anxious). Pay attention to whether your level of fearfulness changes as time goes by. We will be asking you periodically to record your SUDS.

A normal response to exposure therapy is to feel exhausted/tired/some depression after completing an exposure session. Exposure is hard work and your body and mind will be tired out afterwards. If you experience these feelings, it means you are working hard and doing the exposure therapy exactly how you should be! Try and give yourself some self-care afterwards, you deserve it!

The SUDS Scale

0	25	50	75	100
No anxiety<br>Complete calm<br>“relaxing on vacation”	A little “edge”<br>Not bothersome<br>“Ready to go”<br>Anxiety is noticeable	Anxiety bothers you<br>Starts to interfere<br>You would like to reduce the anxiety	Anxiety really bothers you<br>You think about leaving<br>Very interfering	The most anxiety you’ve ever felt or could imagine feeling

You can use any number between 0 and 100.

The anchors above are descriptions to give you a sense of the scale.

Session 1

Safety Behaviours

Safety behaviours are actions carried out with the intention of preventing a feared catastrophe. In the short-term they often give a sense of relief, but in the long-term they are unhelpful because they prevent the disconfirmation of the beliefs that are maintaining anxiety.

What is a catastrophe?

Catastrophes can vary enormously, but tend to be about different kinds of threats to the individual, for example:

- Physical threat - *"I'll be killed", "I'll be hurt"*
- Psychological threat - *"I'll go mad"*, *"I can't cope"*
- Social threat - *"I'll embarrass myself and never be able to show my face again", "They will think I'm an idiot"*
- Eating disorder threat – *"I'll gain weight and never lose it."*

What is your feared catastrophe?

What types of safety behaviours are there?

There are four types of safety behaviours:

1. *Avoidance* - e.g. not going to a feared situation
2. *Escape* - e.g. leaving a feared situation

3. *Eating disorder safety behaviours* - exercising, counting calories, body checking, purging

4. *Subtle avoidance*, which can include things we do in our minds - e.g.

- distraction - counting in my head during a panic to stop myself from going mad
- calming my breathing - otherwise I'll be overwhelmed by my fear and lose control
- averting my eyes - in case someone picks on me and I'm humiliated

What safety behaviours do you use?

What are the effects of safety behaviours?

- *Short term*: In the short-term safety behaviours lead to a reduction in anxiety. Any form of escape or avoidance is often accompanied by a powerful feeling of relief. Relief is powerful negative reinforcer, and once an individual has learned that a safety behaviour leads to relief, they are likely to use it again.
- *Long term*: In the longer term, safety behaviours act to maintain anxiety by preventing the disconfirmation of unhelpful beliefs. For example, if someone has the belief *"dogs will attack me and bite my face"* and avoids dogs, they don't get the opportunity to learn that most dogs are friendly, or fail to learn the difference between friendly and unfriendly dogs.
- *Unintended consequences*: Safety behaviours often have unintended consequences which can reinforce the original belief, make the anxiety worse, or lead to other problems.

What is the difference between a safety behaviour and adaptive behaviour?

Just by looking at the behaviour itself, there is no way of telling the difference between an unhelpful safety behaviour and a helpful adaptive behaviour. It is the *intention* which matters - safety behaviours are those which are *intended* to avert a feared catastrophe. For example, if the strategy of distraction is used to cope with a painful experience (e.g. a visit to the dentist) we say it is helpful. However, if distraction is used with the *intention* of preventing a catastrophe (e.g. to avoid a feeling of panic that I fear will make me go mad) then it can be viewed as an unhelpful safety behaviour

Safety Behaviours and Consequences

Early Experience	Belief	Safety Behaviour	Short Term Consequence	Long Term Consequence	Unintended Consequence
Made fun of for appearance and being exposed to a thin-ideal beauty standard in the media	If I gain weight, I will never be able to lose it, no one will like me, I will be rejected and abandoned	Counting calories (restricting) and other behaviours (over -exercise)	Reduction in anxiety because I am in control of weight gain	No opportunity to learn that I won't be abandoned or rejected if I lose weight, won't learn that I don't need to restrict and engage in compensatory behaviours to not gain weight	Difficulty enjoying eating food and relying on compensatory behaviours which impacts physical health and leads to disordered eating
Physically assaulted by a group of men walking home after I looked at them	People are dangerous, if I make eye contact with others, I will be attacked	Avert my eyes to avoid contact			
Making a mistake on a presentation in school	If I make a mistake everything will be horrible				
Parents getting divorced					

SUDS SHEET - Exposure 1

Date: __________

SUDS 1: ________

SUDS 2: ________

SUDS 3: ________

SUDS 4: ________

SUDS 5: ________

SUDS 6: ________

SUDS 7: ________

SUDS 8: ________

Homework

Complete exposure script and record SUDS; before, during (the highest point) and after

1. Date: _____

SUDS before: _______

SUDS during: ______

SUDS After: _______

2. Date: _____

SUDS before: _______

SUDS during: ______

SUDS After: _______

3. Date: _____

SUDS before: _______

SUDS during: ______

SUDS After: _______

4. Date: _____

SUDS before: _______

SUDS during: ______

SUDS After: _______

5. Date: _____

SUDS before: _______

SUDS during: ______

SUDS After: _______

6. Date: _____

SUDS before: _______

SUDS during: ______

SUDS After: _______

Session 2

Common Responses to Exposure Therapy

Exposure therapy is hard work and your body and mind may be tired out afterwards.

Normal responses to exposure therapy can include:

- Feeling tired
- Feeling exhausted
- Some depression

You may feel some of these things after completing an exposure session. If you experience these feelings, it means you are working hard and doing the exposure therapy exactly how you should be! Try and give yourself some self-care afterwards, you deserve it!

Safety Behaviours

Continue completing the safety behaviours table on page 7 (scenario 2)

Avoidance Hierarchy

Construct a ladder of places or situations that you avoid. At the top of the ladder put those which make you most anxious. At the bottom of the ladder put places or situations you avoid, but which don't bother you as much. In the middle of the ladder put ones that are 'in-between'. Give each item a rating from 0-100% according to how anxious you would feel if you had to be in that situation. Overcome your anxiety by approaching these situations, starting from the bottom of the ladder.

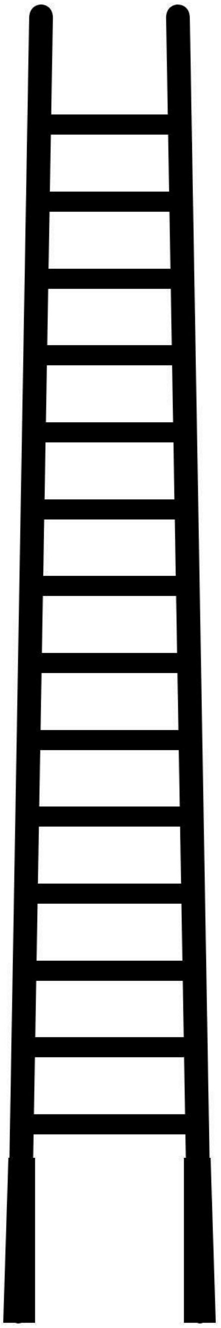

Situation	Anxiety (0-100%)

SUDS SHEET - Exposure 2

Date: ___________

SUDS 1: _________

SUDS 2: _________

SUDS 3: _________

SUDS 4: _________

SUDS 5: _________

SUDS 6: _________

SUDS 7: _________

SUDS 8: _________

Homework

Read exposure script and record SUDS; before, during (the highest point) and after

1. Date: _____

SUDS before: _______

SUDS during: ______

SUDS After: _______

2. Date: _____

SUDS before: _______

SUDS during: ______

SUDS After: _______

3. Date: _____

SUDS before: _______

SUDS during: ______

SUDS After: _______

4. Date: _____

SUDS before: _______

SUDS during: ______

SUDS After: _______

5. Date: _____

SUDS before: _______

SUDS during: ______

SUDS After: _______

6. Date: _____

SUDS before: _______

SUDS during: ______

SUDS After: _______

Session 3

Common Responses to Exposure Therapy

Exposure therapy is hard work and your body and mind may be tired out afterwards.

> Normal responses to exposure therapy can include:
>
> - Feeling tired
> - Feeling exhausted
> - Some depression

You may feel some of these things after completing an exposure session. If you experience these feelings, it means you are working hard and doing the exposure therapy exactly how you should be! Try and give yourself some self-care afterwards, you deserve it!

Safety Behaviours

Continue completing the safety behaviours table on page 7 (scenario 3 and 4)

Avoidance Hierarchy

Review avoidance hierarchy and adapt if appropriate (page 11)

SUDS SHEET – Exposure 3

Date: ___________

SUDS 1: _________

SUDS 2: _________

SUDS 3: _________

SUDS 4: _________

SUDS 5: _________

SUDS 6: _________

SUDS 7: _________

SUDS 8: _________

Homework

Read exposure script and record SUDS; before, during (at the highest point) and after

1. Date: _____

SUDS before: _______

SUDS during: ______

SUDS After: _______

2. Date: _____

SUDS before: _______

SUDS during: ______

SUDS After: _______

3. Date: _____

SUDS before: _______

SUDS during: ______

SUDS After: _______

4. Date: _____

SUDS before: _______

SUDS during: ______

SUDS After: _______

5. Date: _____

SUDS before: _______

SUDS during: ______

SUDS After: _______

6. Date: _____

SUDS before: _______

SUDS during: ______

SUDS After: _______

Session 4 (Final Session)

What I Learned in Exposure Therapy or What I know About Anxiety Now

---

- The more I face something anxiety provoking the easier it gets
- If I feel like avoiding something because it makes me uncomfortable, I should face it instead
- Things that make me uncomfortable/anxious are usually not as bad they seem
- I should let myself feel my emotions
- Safety behaviors work in the short, but not the long term
- If you stay in a situation that is uncomfortable long enough, eventually it will not be uncomfortable
- Things that I am afraid of happening are likely not as bad as I think or are less likely to happen than I think
- I can handle my anxiety and my eating disorder!
- Other things I have learned:

____

____

____

____

____

____

____

____

____

____

____

SUDS SHEET – Exposure 4

Date: ___________

SUDS 1: _________

SUDS 2: _________

SUDS 3: _________

SUDS 4: _________

SUDS 5: _________

SUDS 6: _________

SUDS 7: _________

SUDS 8: _________
